# A Comparison of In-Person and Online Training in a Statewide Clinical Education Program for Dissemination of HIV, HCV and STD Clinical Evidence

**DOI:** 10.3233/SHTI190756

**Published:** 2019-08-21

**Authors:** Dongwen Wang

**Affiliations:** Department of Biomedical Informatics, Arizona State University, Scottsdale, AZ, USA

**Keywords:** Internet training, teacher training, knowledge

## Abstract

We compared in-person and online training in a statewide education program to disseminate HIV, HCV, and STD clinical evidence. In a study period of three months, 250 clinicians completed four training courses delivered in dual formats. Course evaluation was positive regarding useful information, easy comprehension, knowledgeable trainer, appropriate format, knowledge increase, intention to use knowledge, and plan to change practice. Online training became a preferred format by clinicians when compared to in-person training (p=0.01).

## Introduction

The pandemic of HIV infection has been a major public health challenge for decades. Hepatitis C virus (HCV) infection has also increased rapidly in recent years. Co-infection of HIV, HCV, and other sexually transmitted diseases (STDs) such as syphilis and gonorrhea is frequently reported. With the frequent updates of clinical evidence on HIV, HCV, and STD, timely and effective dissemination of the latest research to community healthcare providers is crucial for translating medical knowledge into routine patient care [1–2].

Since the early 1990s’, the New York State Department of Health AIDS Institute has been sponsoring the Clinical Education Initiative (CEI) program for community healthcare providers serving HIV, HCV, and STD patients [3]. In the early years, the CEI program utilized a traditional, in-person approach for training by sending domain experts across the state to give lectures or workshops. This in-person education program successfully trained thousands of clinicians. With the development of information and communication technologies, CEI initiated its online training program in 2008, aiming to complement and to integrate with in-person education. Since then, CEI developed hundreds of multimedia learning modules, online continuing medical education (CME) and continuing nursing education (CNE) courses, interactive case simulation tools, and other digital resources [4–5]. These resources were disseminated through the web, mobile apps, email newsletters, and online social networks to thousands of clinicians from 170+ countries over the world [6]. The participating clinicians had very positive evaluations of the CEI online training program [7–9].

Compared to traditional in-person continuing professional education, online training is advocated for wide geographical availability, rapid outreach to the target audience, flexibility in resource access and use, as well as cost-efficiency [10–11]. Yet studies to compare online and in-person training directly are scarce and inconclusive.

Here we report a study to compare online and in-person training within the New York State CEI program. The results from this study can help to compare online and in-person cross-disciplinary continuing professional education for HIV, HCV, and STD clinicians, and to guide the future design of education programs for more effective dissemination of clinical evidence to community healthcare providers.

## Methods

We conducted the study in a period from April 1, 2015, to June 30, 2015. Among the hundreds of courses and training sessions offered by the CEI program, we selected for analysis only those delivered during the study period in both online and in-person formats to clinicians. For a traditional in-person training session, a clinician in the community first participated in a lecture or workshop delivered by a domain expert, and then logged on to the CEI Student Portal online system to complete a course evaluation. For an online course, a clinician logged on to the CEI Student Portal system, selected a specific course, reviewed the multimedia course materials, and then completed the same course evaluation as for in-person training [9].

For study measures, we focused on the training content, format, participants’ improvement in knowledge, and impact on their clinical practice. Specifically, we collected the following self-reported data through course evaluation: (1) usefulness and relevance of the information in a training course; (2) easiness of comprehension; (3) expertise/knowledge of trainer/speaker; (4) appropriateness of training format; (5) participant’s knowledge of the clinical topic before and after the training; (6) intention to use the knowledge learned; and (7) plan to change clinical practice after training. We used a five-point Likert-scale measure (*strongly agree*, *agree*, *neutral*, *disagree*, and *strongly disagree*), which were further grouped into positive (*strongly agree* and *agree*) and non-positive (*neutral*, *disagree*, and *strongly disagree*) responses for data analyses on items (1), (2), (3), and (6). For item (5), we used five discrete levels to indicate a clinician’s knowledge on a specific training topic (*novice*, *not very knowledgeable*, *knowledgeable*, *very knowledgeable*, and *expert*) before and after the training, and then calculated the difference (*at least one level increase* vs. no *increase*) for data analysis. Additional details of CEI program evaluation can be found in our previous publications [7–9].

We used the SPSS package for statistical analysis [12]. We compared the proportions of positive responses between in-person and online training. We used the chi-square test to examine the statistical significance of differences.

## Results

During the study period, four training courses were delivered in dual formats to 250 clinicians (online 131, in-person 119). These four courses were: (1) *STD-HIV Inter-Relationship* (81 completions); (2) *Treatment for Hepatitis C: New Tests, New Drugs & New Recommendations* (91 completions); (3) *Vaginitis* (32 completions); and (4) *The Clinical Diagnosis and Treatment of Syphilis* (46 completions).

Clinicians’ course evaluation was positive for both in-person and online training regarding useful information (in-person 96%, online 92%), easiness of comprehension (in-person 91%, online 93%), knowledgeable trainer (in-person 97%, online 90%), and appropriate format (in-person 74%, online 87%). In terms of the impact, we found: (1) 56% clinicians participated in in-person training and 43% in online training reported at least one level increase in knowledge; (2) 87% clinicians in in-person training and 86% in online training intended to use the learned knowledge; and (3) 39% in in-person training and 22% in online training planned to change practice. In-person training recorded better evaluation in terms of knowledgeable trainer (p=0.04), improvement of knowledge (p=0.03), and change of practice (p<0.01). Online training recorded better evaluation in terms of appropriate format (p=0.01). No significant difference was found in terms of useful information (p=0.25), easy comprehension (p=0.49), and intention to use knowledge (p=0.79). The detailed evaluation data are shown in Table 1.

## Conclusions

Both online and in-person training can effectively disseminate HIV, HCV, and STD clinical evidence to community healthcare providers. Although online training is still not equal to in-person training in certain aspects, it has become a preferred format by clinicians to update their knowledge. Future research is required: (1) to verify the generalizability of the findings from this study; and (2) to combine the features of online and in-person training to better address clinicians’ needs and preferences for more effective knowledge dissemination.

## Figures and Tables

**Table 1 –:** Evaluation of In-Person and Online Training

Measures	In-Person (n=119)	Online (n=131)	Total (n=250)
Information Useful and Relevant			
*Positive*	114 (96%)	121 (92%)	235 (94%)
*Non-Positive*	5 (4%)	10 (8%)	15 (6%)
Easy to Comprehend			
*Positive*	108 (91%)	122 (93%)	230 (92%)
*Non-Positive*	11 (9%)	9 (7%)	20 (8%)
Knowledgeable Trainer*			
*Positive*	115 (97%)	118 (90%)	233 (93%)
*Non-Positive*	4 (3%)	13 (10%)	17 (7%)
Format Appropriate*			
*Positive*	88 (74%)	114 (87%)	202 (81%)
*Non-Positive*	31 (26%)	17 (13%)	48 (19%)
Improvement of Knowledge*			
*Positive*	67 (56%)	56 (43%)	123 (49%)
*Non-Positive*	52 (44%)	75 (57%)	127 (51%)
Intend to Use Knowledge			
*Positive*	104 (87%)	113 (86%)	217 (87%)
*Non-Positive*	15 (13%)	18 (14%)	33 (13%)
Will Change Practice*			
*Positive*	46 (39%)	29 (22%)	75 (30%)
*Non-Positive*	73 (61%)	102 (78%)	175 (70%)

* Significant difference (p<0.05) between in-person and online training detected
